# Bradykinin Inhibits Oxidative Stress-Induced Cardiomyocytes Senescence via Regulating Redox State

**DOI:** 10.1371/journal.pone.0077034

**Published:** 2013-10-25

**Authors:** Ruolan Dong, Xizhen Xu, Geng Li, Wenjing Feng, Gang Zhao, Junjie Zhao, Dao Wen Wang, Ling Tu

**Affiliations:** 1 Department of Geriatric Medicine, Tongji Hospital, Tongji Medical College, Huazhong University of Science and Technology, Wuhan, People's Republic of China; 2 The Institute of Hypertension and Department of Internal Medicine, Tongji Hospital, Tongji Medical College, Huazhong University of Science and Technology, Wuhan, People's Republic of China; University of Pecs Medical School, Hungary

## Abstract

**Background:**

Cell senescence is central to a large body of age related pathology, and accordingly, cardiomyocytes senescence is involved in many age related cardiovascular diseases. In consideration of that, delaying cardiomyocytes senescence is of great importance to control clinical cardiovascular diseases. Previous study indicated that bradykinin (BK) protected endothelial cells from senescence induced by oxidative stress. However, the effects of bradykinin on cardiomyocytes senescence remain to be elucidated. In this study, we investigated the effect of bradykinin on H_2_O_2_-induced H9C2 cells senescence.

**Methods and Results:**

Bradykinin pretreatment decreased the senescence induced by H_2_O_2_ in cultured H9C2 cells in a dose dependent manner. Interestingly, 1 nmol/L of BK almost completely inhibited the increase in senescent cell number and p21 expression induced by H_2_O_2_. Since H_2_O_2_ induces senescence through superoxide-induced DNA damage, we also observed the DNA damage by comet assay, and BK markedly reduced DNA damage induced by H_2_O_2_, and moreover, BK treatment significantly prevented reactive oxygen species (ROS) production in H9C2 cells treated with H_2_O_2_. Importantly, when co-incubated with bradykinin B2 receptor antagonist HOE-140 or eNOS inhibitor N-methyl-L-arginine acetate salt (L-NAME), the protective effects of bradykinin on H9C2 senescence were totally blocked. Furthermore, BK administration significantly prevented the increase in nicotinamide adenine dinucleotide phosphate (NADPH) oxidase activity characterized by increased ROS generation and gp91 expression and increased translocation of p47 and p67 to the membrane and the decrease in superoxide dismutase (SOD) activity and expression induced by H_2_O_2_ in H9C2 cells, which was dependent on BK B2 receptor mediated nitric oxide (NO) release.

**Conclusions:**

Bradykinin, acting through BK B2 receptor induced NO release, upregulated antioxidant Cu/Zn-SOD and Mn-SOD activity and expression while downregulating NADPH oxidase activity and subsequently inhibited ROS production, and finally protected against cardiomyocytes senescence induced by oxidative stress.

## Introduction

Aging has been defined in many different ways, since people began to study up on this subject. From a biological point of view, aging is an universal, progressive, and irreversible decline of function over time, resulting in reduction of cell function and eventually cell death [1]. Aging-related biochemical and cell-biological changes of cardiomyocytes lead to pathophysiological conditions, especially reduced heart function and heart diseases [2]. Consequently, delaying cell senescence is a promising strategy in maintaining and promoting myocardial function.

It is previously shown that cell senescence originates from DNA damage. When telomeres shorten beyond a certain threshold, cells become unstable, undergo senescence and lose their ability to divide [3]. In pathological conditions, senescence is a state of permanent cell growth arrest [4] ahead of schedule that can be induced by DNA damage by exposure to various insults, such as reactive oxygen species (ROS) [5]. Besides, it can also be induced by other stress or injuries, such as the loss of bioavailable nitric oxide generation and downregulation of bradykinin type 2 receptor expression [6]. Senescent cells show declined function, when accumulates to some extent, some age related diseases may emerge. Previous data indicated that cardiomyocytes senescence contributed to cardiovascular diseases (CVD). Senescent cardiomyocytes show deleterious changes in function [3], which are reminiscent of the aberrant myocardium changes observed in aging and heart failure [7]. Furthermore, an increase of senescent cardiomyocytes and senescence markers are observed in the myocardium of aged rodents with myocardial dysfunction [3].

Angiotensin-converting enzyme inhibitors (ACEI) play important roles in the treatment of many age-related cardiovascular diseases [7]–[8]. The therapeutic effect of ACEI involves lots of mechanisms, one of which is the increase of bradykinin, member of the kallikrein-kinin system (KKS) [9]. KKS is a complex multi-enzyme system composed of circulating and tissue kallikrein and kinin. It is well established that tissue kallikrein and kinin play crucial and diverse roles in cardiovascular and renal homeostasis [10]. Our previous data also show that kallikrein gene delivery reduces blood pressure and ameliorates renal function in spontaneously hypertensive and 5/6 nephrectomized rats [11]. Bradykinin is a principal active agent of KKS, stimulated by many kinds of inflammatory insults and injures. BK exerts its major effects through the activation of two bradykinin receptors (BKRs): B1R and B2R [12]. Previous studies demonstrated that BK protects endothelial cells from oxidative stress-induced senescence through the release of nitric oxide, which is mediated by B2 receptor activation [2]. In contrast, aging is associated with a decreased availability of cardiac BK B2 receptor [13]. BK B2 receptor knockout in diabetic mice leads to an accelerating aging phenotype, due to an increase of oxidative stress [14]. These data suggest that bradykinin and its receptor dysfunction is associated with aging. However, whether BK can protect cardiomyocytes from oxidative stress-induced senescence remains unclear. Hence, we evaluated the effect of BK pretreatment on H9C2 cells senescence induced by oxidative stress and investigated the potential mechanisms.

## Materials and Methods

### Reagents

Streptomycin sulfate, penicillin, Dulbecco's modified Eagle's medium (DMEM) and fetal bovine serum (FBS) were purchased from Gibco. Bradykinin (BK), B2R antagonist HOE-140 and eNOS inhibitor N^ω^-methyl-L-arginine acetate salt (L-NAME) were from Sigma-Aldrich. 2,7-Dichlorofluorescein diacetate (H2CFDA) was also obtained from Sigma-Aldrich. Caspase-3 colorimetric assay kit and comet assay kit were obtained from Sigma-Aldrich. β-gal staining kit was from Invitrogen. The primary antibodies against Cu/Zn SOD and Mn SOD and p21 and p67-phox were obtained from Santa Cruz Biotechnology. The primary antibody against p47-phox, gp91-phox, p21 and B2R were from epitomic. Secondary antibodies (goat anti-rabbit and goat anti-mouse horseradish peroxidase-conjugated secondary antibodies) were from Sigma-Aldrich. The superoxide anion detection kit was obtained from beyotime. All other chemicals and reagents were purchased from Sigma-Aldrich unless otherwise specified.

### Cell culture

Rat cardiomyocytes cell line H9C2 was purchased from the American Type Culture Collection. H9C2 were cultured in growth medium DMEM (containing streptomycin sulfate, penicillin) containing 10% heat-inactivated FBS in an incubator at an atmosphere of 5% CO_2_, 95% air at 37°C. Cells were passaged at a 1∶3 ratio when they reached confluence. Experiments were conducted on cells between passages 4 and 10.

### ROS-induced senescence and pharmacological treatments

H9C2 cells, 24 hours after seeding in 12-well plates, were exposed to various concerntrations of H_2_O_2_ to induce DNA damage and senescence. After 2 hours, the medium was replaced with normal medium. BK with different kinds of concentrations was given 30 minutes before the induction of senescence. The B2R antagonist HOE-140 (1 µmol/l) and eNOS inhibitor N^ω^-methyl-L-arginine acetate salt (L-NAME) (10 µmol/l) were added 5 minutes before BK treatment, after which BK was added without replacing the medium. 24 hours after H_2_O_2_ treatment, H9C2 cells were harvested for senescence determination. At least 3 separate experiments were performed per treatment.

### Senescence-associated β-galactosidase (sa-β-gal) staining

To evaluate the number of senescent cells, we performed the sa-β-gal staining. According to the method described before [15], cells were incubated in freshly prepared staining solution for 24 h at 37°C. Light microscopic pictures were taken on an inverted microscope with a 200 magnification, and the number of senescent cells was counted per microscopic field. In each well, 5 random fields were evaluated.

### Comet Assay

H9C2 cells were treated as described before with H_2_O_2_ and BK, and 24 hours after treatment, DNA damage was determined with a commercially available kit for single cell electrophoresis, the so-called Comet Assay, and the detailed protocol was described as before [16].

### Caspase-3 activity assay

Caspase-3 activity was determined using the ApoAlert Caspase Colorimetric Assay kit in accordance with the manufacturer's protocol. In brief, at least 1×10^6^ cells per sample were lysed in 50 µl of lysis buffer, and the protein concentrations in the samples were estimated using the Bio-Rad protein assay. After incubation on ice for 10 minutes, the samples were centrifuged at 16,000×g for 3 min at 4°C. Each supernatant was mixed with 50 µl 2× Reaction Buffer/DTT mix and 5 µl of 1 mM Caspase-3 Substrate (DEVD-pNA, 50 µM final concentration), and the samples were then incubated for 1 h at 37°C in the dark. Developed colour was measured at 405 nm, and caspase-3 activity was calculated in terms of absorbance units per µg protein.

### Flow Cytometric Evaluation of Intracellular ROS

The ROS generation was monitored by flow cytometry using peroxide-sensitive fluorescent probe 2,7-dichlorofluorescein diacetate (DCFH-DA) [17]. DCFH-DA is converted by intracellular esterases to DCFH, which is oxidized into the highly fluorescent dichlorofluorescein (DCF) in the presence of a proper oxidant. The cells were loaded with 10 µmol/L DCFH-DA in serum-free medium at 37°C for 30 min and then washed twice with PBS. Next, the cells were monitored with a flow cytometer (BD, U.S.A.) at an excitation wavelength of 488 nm and an emission wavelength of 525 nm. ROS was determined by comparing the changes in fluorescence intensity to those of the control.

### Measurement of superoxide

NADPH oxidase activity was assessed by measuring superoxide production according to the manufacturer's instructions. In addition, NADPH oxidase activity was also evaluated using lucigenin method as previously described [18].

### SOD activity determination

SOD activity in H9C2 cells homogenates was determined using spectrophotometry [19], according to the manufacture's instructions (Jiancheng Bioengineering Institute, Nanjing, China).

### Western blotting

To prepare whole cell extracts, cells were washed with PBS and lysed for 30 min on ice in hypotonic lysis buffer (10 mmol/L Tris, pH 7.4; 0.5 mmol/L EDTA; 1 mmol/L sodium orthovanadate; 1 mmol/L phenylmethylsulfonyl fluoride; 10 µg/ml leupeptin; 10 µg/ml aprotinin). The lysates were centrifuged at 12,000× g for 20 min at 4°C, and the supernatant was obtained as total protein. Membrane protein was extracted as previously described [20]. In brief, the lysates were centrifuged at 400,000 g for 30 min. The resulting supernatant was harvested (cytosolic fraction), and the pellet was resuspended in hypotonic buffer containing 1% Triton X-100. This mixture was re-centrifuged at 400,000 g for 30 min, and the supernatant (membrane fraction) was collected. The supernatants were separated on 10% SDS-polyacrylamide gels. The loaded amounts of extract corresponded to equal cell quantities. After electrophoresis, the proteins were transferred to PVDF membranes by wet electroblotting. The membranes were blocked with 5% nonfat dry milk in Tris-buffered saline and then incubated with primary antibodies overnight at 4°C. Blots were developed with peroxidase-conjugated secondary antibody, and proteins were visualized by enhanced chemiluminescence (Pierce, Rockford, IL).

### Statistical analysis

Continuous data were expressed as means ± S.E.M. Comparisons between groups were performed by a one-way analysis of ANOVA with post hoc analyses performed using the Student-Newman-Keuls method. A *p* value <0.05 was considered significant.

## Results

### Induction of H9C2 senescence

**Figure 1:**
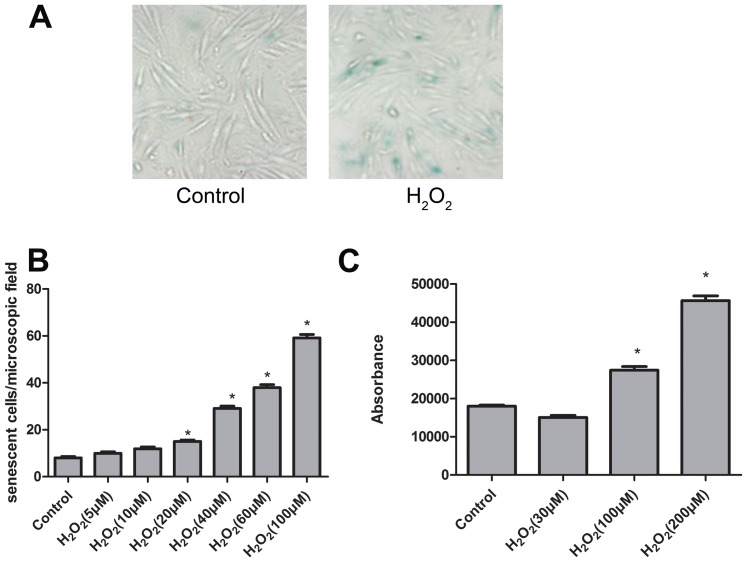
Low dose of H_2_O_2_ induced senescence in H9C2 cells without causing apoptosis. A, Representative pictures of sa-gal-stained H9C2 cells. Left, control (without H_2_O_2_); right, 3 days after stimulation with 30 µmol/L of H_2_O_2_ for 2 hours. B, Number of sa-gal–positive cells per microscopic field 3 days after a 2-hour H_2_O_2_ (0–100 µmol/L) treatment. Bars represent means±SEM (*P<0.05 vs control; n = 3 experiments). C, Caspase-3 activity, measured as luminescence, in H9C2 3 days after treatment with H_2_O_2_ (0–200 µmol/L) for 2 hours. Bars represent means±SEM (*P<0.05 vs control; n = 3 experiments).

H9C2 cells were exposed to various concentrations of H_2_O_2_ ranging from 30–200 µmol/L for 2 hours and subsequently cultured for 3 days, after which senescence and apoptosis were determined. We successfully induced the senescence of H9C2 with 30 µmol/L of H_2_O_2_ (Figure 1A). The number of senescent cells is increased in a dose-dependent manner (Figure 1B), However, it led to apoptosis when exposed to the concentrations of H_2_O_2_ more than 100 µmol/L(Figure 1C). Intriguingly, 30 µmol/L of H_2_O_2_ had no apparent effect on caspase-3 activity (Figure 1C). Therefore, additional experiments were performed with 30 µmol/L of H_2_O_2_.

### BK protects against H9C2 cell senescence

**Figure 2:**
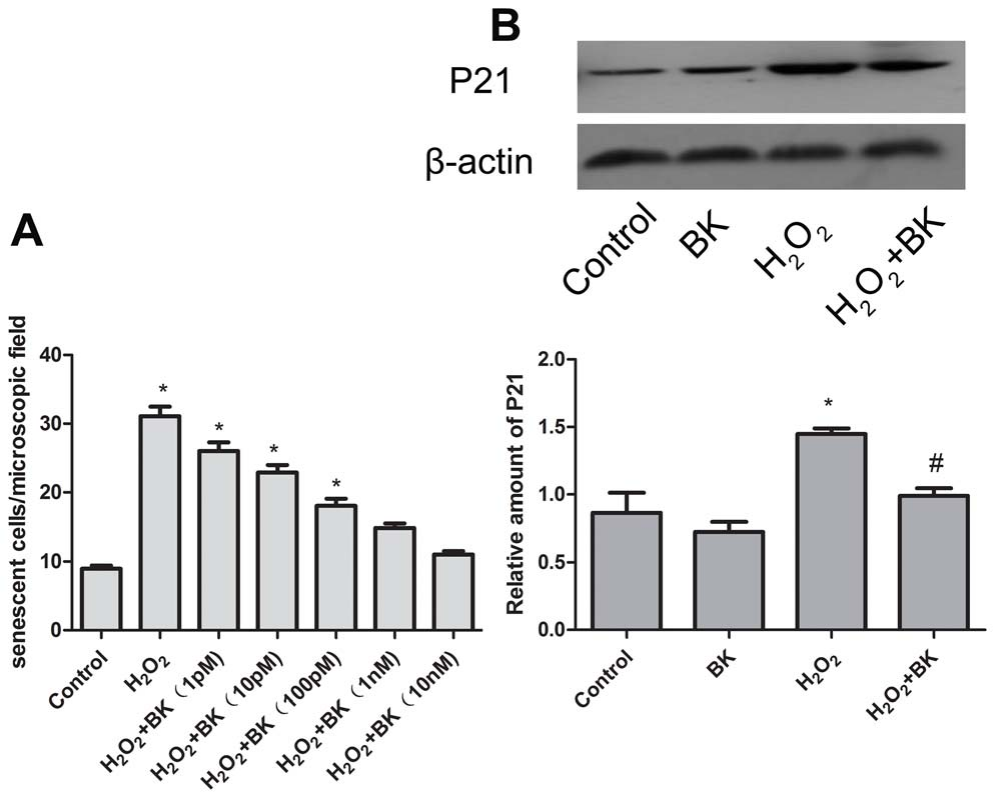
Effects of bradykinin pretreatment on H_2_O_2_-induced H9C2 cells senescence. A, number of senescent cells 3 days after treatment. Bars represent means±SEM (*P<0.05 vs control; n = 6 experiments). B, p21 protein levels 24 hours after treatment. Bars represent means ±SEM (*P<0.05 vs control; ^#^ *P*<0.05 vs H_2_O_2_; n = 3 experiments).

To study the effect of BK on cardiomyocytes senescence induced by H_2_O_2_, BK B2 receptor expression in H9C2 cells was determined. As shown in Figure S1 and Figure S2, BK B2 receptor was expressed in H9C2 cells, and H_2_O_2_ caused significant reduction in B2 receptor expression, and moreover BK administration apparently prevented the decrease in B2 receptor expression induced by H_2_O_2_. Importantly, HOE-140 and L-NAME totally abolished the inhibitory effects of BK on B2 receptor expression. H9C2 cells were pretreated with different concentrations of BK (1 pmol/L–10 nmol/L) prior to 30 µmol/L of H_2_O_2_ treatment. BK decreased the number of senescent cells in a dose dependent manner (Figure 2A). 1 nmol/L of BK almost completely abrogated H_2_O_2_ induced H9C2 cell senescence. However, BK had no effects on H9C2 cells that were not treated with H_2_O_2_.

p21 is an important mediator of cell senescence [21]. As a secondary variable to assess senescence, p21 expression was measured by Western blot. p21 level was significantly increased 24 hours after treatment with 30 µmol/L of H_2_O_2_ (Figure 2B). Importantly, BK administration significantly prevented p21 upregulation induced by H_2_O_2_ in H9C2 cells. Importantly, HOE-140 and L-NAME totally abolished the inhibitory effects of BK on p21 expression as shown in Figure S1 and Figure S2. Collectively, these data indicated that BK markedly proteced against H9C2 cells senescence induced by H_2_O_2_.

### BK protects against DNA damage in H9C2 cells

**Figure 3:**
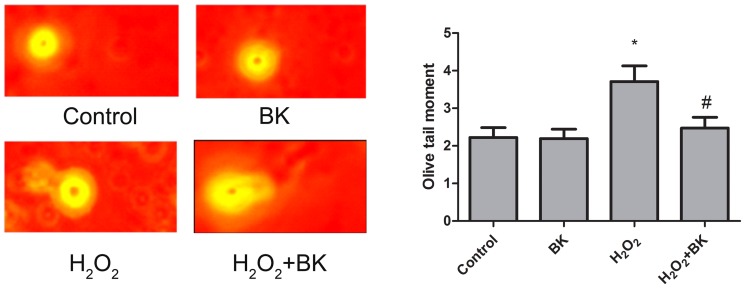
Effects of BK on DNA damage in H9C2 cells treated with H_2_O_2_. A, Left, control (without H_2_O_2_); right, 24 hours after stimulation with 30 µmol/L of H_2_O_2_ for 2 hours. Olive tail moment is calculated as a percentage of DNA in the tail distance to center of gravity of tail. Bars represent means ±SEM (*P<0.05 vs control; ^#^ *P*<0.05 vs H_2_O_2_; n = 3 experiments).

Bradykinin protects against cardiomyocyte senescence in many aspects, one of which is just the defence against DNA damage [22]. Therefore, we measured the olive tail moment in a comet assay 24 hours after treatment of 1 nmol/L of BK. As expected, H_2_O_2_ induced the olive tail moment formation (Figure 3), indicating an increase in DNA damage. Interestingly, BK treatment prevented increased DNA damage induced by H_2_O_2_ treatment (Figure 3). These data suggested that BK inhibited DNA damage induced by H_2_O_2_ in H9C2 cells.

### BK protects against H9C2 cells senescence through BK B2 receptor-mediated NO release

**Figure 4:**
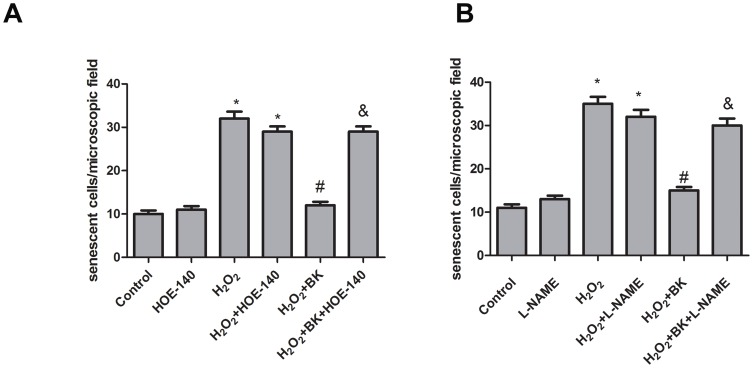
Effects of BK B2 receptor antagonist and eNOS inhibitor on the protective effect of BK against H9C2 cells senescence. Senescence was measured as the number of sa-β-gal–positive cells 3 days after treatment with H_2_O_2_. A, effect of BK B2 receptor antagonist HOE-140 on the protective effect of BK against H9C2 cells senescence. B, effect of L-NAME on the protective effect of BK against H9C2 cells senescence. Bars represent means ±SEM (*P<0.05 vs control; ^#^ *P*<0.05 vs H_2_O_2_; ^&^ *P*<0.05 vs H_2_O_2_+BK; n = 6).

Bradykinin is a principal active agent of KKS, stimulated by many kinds of inflammatory insults and injures, and BK exerts its major effects through the activation of two bradykinin receptors: BK-1R and BK-2R [12]. Previous study indicated that most of the protective effects of bradykinin are mediated through activation of B2 receptor. To explore whether the protective effect against H9C2 cells senescence is also dependent of BK B2 receptor, BK B2R antagonist HOE-140 was used, and moreover HOE-140 fully antagonized the protective effect of BK on H9C2 cells senescence. While being without effect on cells that were not treated with H_2_O_2_ (Figure 4A). It was previously shown that BK inhibited endothelial cells senescence induced by H_2_O_2_ treatment through NO release, therefore, in this study, we investigated the possible effect of NO production on BK mediated inhibitory effects on H9C2 cells senescence. As expected, L-NAME administration abrogated the protective effect of BK on H9C2 cells senescence (Figure 4B). Taken together, we concluded that BK protects against H9C2 cells senescence through stimulation of BK B2 receptor mediated NO release.

### BK protects against cell senescence through regulating redox state

**Figure 5:**
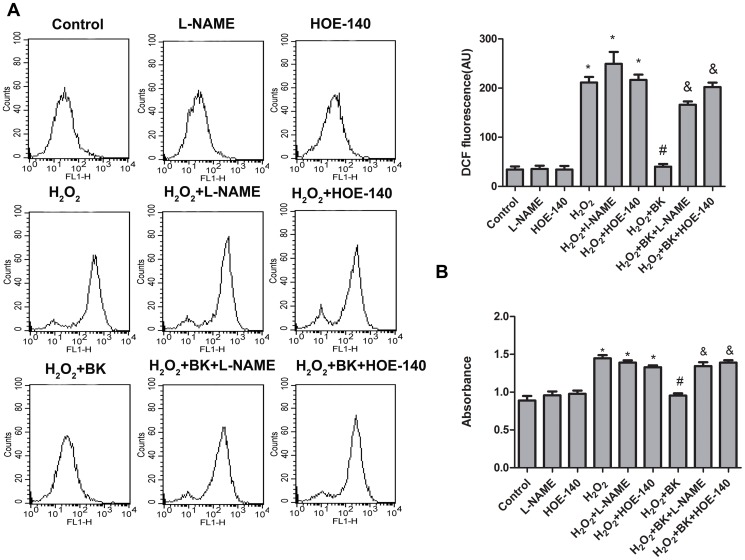
Effects of BK on ROS production in H9C2 cells treated with H_2_O_2_. A, ROS generation was measured by the DCF fluorescence intensity. B, Superoxide generation was measured by the commercial available kit. Bars represent means ±SEM (*P<0.05 vs control; ^#^ *P*<0.05 vs H_2_O_2_; ^&^ *P*<0.05 vs H_2_O_2_+BK; n = 6).

At the cellular level, senescence is a state of permanent growth arrest [23]. Free radical-dependent cellular damage theory was developed since 1956 [24] and remains the theory basis of aging, and importantly oxidative stress induces cellular senescence [25]. Superoxide dismutase (SOD), along with other enzymatic and non-enzymatic anti-oxidants, plays a pivotal role in preventing cellular damage caused by ROS [26]. In this study, we determined the production of ROS by using two different methods. As expected, treatment with H_2_O_2_ caused significant increase in intracellular ROS level in H9C2 cells, and BK administration inhibited ROS production induced by H_2_O_2_, furthermore, administration of HOE-140 and L-NAME completely abrogated the inhibitory effects of BK on ROS production in H9C2 cells as shown in Figure 5A and 5B. These data demonstrated that BK treatment prevented ROS production induced by H_2_O_2_ through BK B2 mediated NO release.

**Figure 6:**
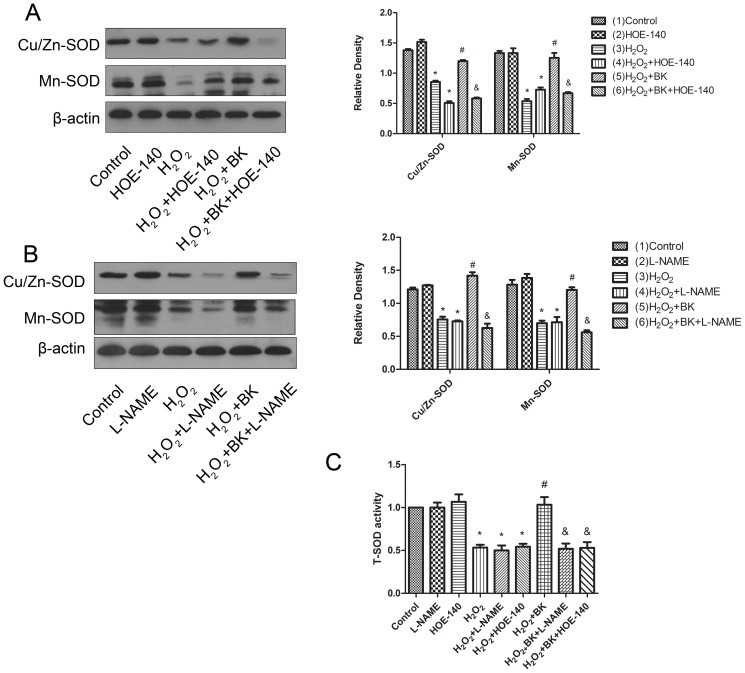
Effects of BK on SOD expression and activity in H9C2 cells treated with H_2_O_2_. A, Effects of HOE-140 on the beneficial effects of BK on SOD expression in H9C2 cells treated with H_2_O_2_. B, Effects of L-NAME on the beneficial effects of BK on SOD expression in H9C2 cells treated with H_2_O_2_. C, Effects of HOE-140 and L-NAME on the beneficial effects of BK on total SOD (T-SOD) activity in H9C2 cells treated with H_2_O_2_. Bars represent means ±SEM (*P<0.05 vs control; ^#^ *P*<0.05 vs H_2_O_2_; ^&^ *P*<0.05 vs H_2_O_2_+BK; n = 3 experiments).

**Figure 7:**
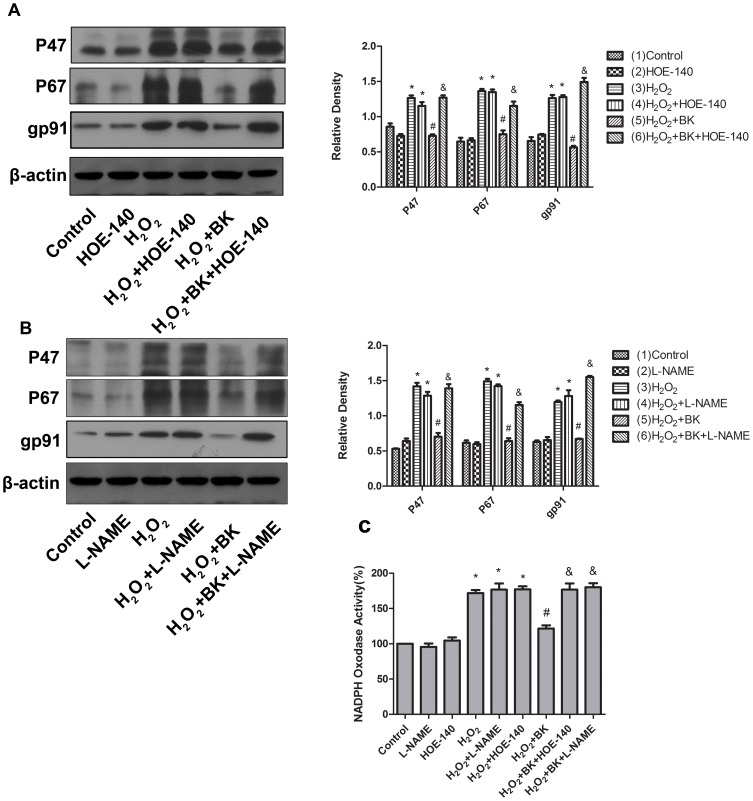
Effects of BK on membrane p47, p67 and gp91 expression and NADPH oxidase activity in H9C2 cells treated with H_2_O_2_. A, Effects of BK on membrane p47, p67 and gp91 expression in H9C2 cells treated with H_2_O_2_ and HOE-140. B, Effects of BK on membrane p47, p67 and gp91 expression in H9C2 cells treated with H_2_O_2_ and L-NAME. C, Effects of BK on NADPH oxidase activity in H9C2 cells treated with H_2_O_2_. Bars represent means ±SEM (*P<0.05 vs control; ^#^ *P*<0.05 vs H_2_O_2_; ^&^ *P*<0.05 vs H_2_O_2_+BK; n = 3 experiments).

Cellular ROS levels are temporally and spatially regulated by the fine-tuned balance between the ROS generation system and anti-oxidant enzymes. In the present study, we investigated the activity of SOD and NADPH oxidase in H9C2 cells treated with H_2_O_2_. Interestingly, treatment of H_2_O_2_ caused a significant downregulation in Cu/Zn-SOD and Mn-SOD expression in total protein extract of H9C2 cells as shown in Figure 6A and Figure 6B, and furthermore a significant decrease in T-SOD activity (Figure 6C). In contrast, H_2_O_2_ treatment increased membrane p47, p67 and gp91 expression (Figure 7A and 7B) as well as NADPH oxidase activity (Figure 7C) in H9C2 cells, which indicated that H_2_O_2_ treatment promoted p47 and p67 translocation from cytoplasm to membrane. Importantly, BK treatment prevented the downregulation in Cu/Zn-SOD and Mn-SOD expression and the decrease in T-SOD activity in H9C2 cells treated with H_2_O_2_ as shown in Figure 6A, Figure 6B and Figure 6C. Meanwhile, BK administration markedly inhibited the upregulation in membrane p47, p67 and gp91 expression and the increase in NADPH oxidase activity induced by H_2_O_2_ as shown in Figure 6A, Figure 6B and Figure 6C. As expected, HOE-140 or L-NAME treatment totally abrogated the beneficial effects of BK on SOD and NADPH oxidase activity regulation induced by H_2_O_2_ in H9C2 cells (Figure 6 and Figure 7). Collectively, these data indicated that BK treatment significantly upregulated SOD expression and activity and inhibited NADPH oxidase activity through B2 receptor mediated NO release in H9C2 cells treated with H_2_O_2_.

## Discussion

In the present study, we demonstrated that exogenous BK administration markedly inhibited H9C2 cells DNA damage and subsequent senescence induced by H_2_O_2_. Furthermore, we also provided evidence that exogenous BK prevented H9C2 cells senescence via regulation of redox state, which is characterized by decreased NADPH oxidase activity and increased SOD expression and activity in H9C2 cells treated with H_2_O_2_. Importantly, exogenous BK regulated H9C2 cells redox state through B2 receptor mediated NO release. These data indicated that BK inhibited H9C2 cells senescence via regulation of redox state, which was involved in B2 receptor mediated NO release.

This study showed that exogenous BK protects against ROS-induced DNA damage and cardiomyocytes senescence in a dose–dependent manner. P21, a cyclin-dependent kinase 1A inhibitor, is usually activated by DNA damage [16]. In this study, BK treatment significantly prevented p21 upregulation induced by H_2_O_2_, and prevented DNA damage induced by H_2_O_2_ in H9C2 cells, indicating that BK inhibited oxidative stress mediated senescence induced by H_2_O_2_ in H9C2 cells. Previous data indicated that oxidative stress appears to play an important role in the process of premature aging. However, this process is apparently regulated by the kallikrein-kinin system [21], not only in the cardiovascular system, but also in a broader perspective, and this protective role of the kallikrein-kinin system seems to depend on protection against ROS-induced DNA damage, by regulating redox state, as witnessed by the comet assay in this study.

Previous data indicated that bradykinin B2 receptor promoted endothelial NO synthase activation and protected against cell senescence in endothelial cells [27]. Bradykinin caused concentration-dependent release of NO in many cell types [28]. Emerging evidence revealed that phosphorylation of eNOS and the concurrent increase of NO production contributed significantly to the inhibition of cardiomyocytes apoptosis in the ischemia reperfusion condition [29]. Tissue kallikrein through kinin B2 receptor and NO formation improved cardiac function, limited left ventricular remodeling after myocardial ischemia/reperfusion injury by suppression of oxidative stress [30], and in contrast, shortage of NO and production of ROS are also responsible for ischemia reperfusion injury in heart [31]. Accumulated oxidative damage has been postulated as an important cause of aging [32]. Senescent cells are irreversibly growth-arrested and contain elevated levels of p21 and p16 cyclin-dependent kinase inhibitors [33]. P53 is also activated by DNA damage, which, in turn, causes upregulation of BK B2 receptor [34]. p53 plays an important role in the regulation of p21 expression and senescence [34]–[35]. BK B2 receptor activity increases, serving as an endogenous protective feedback mechanism against further ROS-induced DNA damage and, thus, reducing p21-mediated cellular senescence. The NO release that BK B2 receptor induces may be paid attention to in this process [36]. NO has been described to protect against replicative senescence, effects that are associated with telomeric DNA repair by telomerase [37]. In this study, BK significantly prevented DNA damage induced by H_2_O_2_, which indicated that BK mediated protection against the degradation of DNA, which might involve various DNA repair systems [38], such as the double-strand break repair mechanism, which is shown to mediate the protective effect of statins against cardiomyocyte cell senescence. Importantly, B2 receptor antagonist HOE-140 and eNOS inhibitor L-NAME totally abrogated the inhibitory effects of BK on H9C2 cells senescence induced by H_2_O_2_. Therefore, our study revealed that exogenous bradykinin protected against ROS-induced H9C2 cells senescence through BK B2 receptor mediated NO release.

Free-radical theory of aging proposes that degenerative senescence is largely dependent on the accumulative effect of ROS. The prototypic NADPH oxidase complex consists of a membrane bound p22-phox and one gp91-phox subunit and four cytosolic subunits (p40-phox, p47-phox, p67-phox, and Rac-1). Sustained activation of NAPDH oxidase involves translocation of the cytosolic subunits as well as an increased oxidase subunits expression [39]. In this study, we examined ROS production in H9C2 cells treated with H_2_O_2_. H_2_O_2_ treatment caused increased production of ROS, and BK treatment prevented ROS production,which was associated with decreased NADPH oxidase activity and increased SOD expression and activity dependent on B2 receptor mediated NO release. These data indicated that BK prevented H9C2 cells senescence induced by H_2_O_2_ through reducing ROS generation and increasing ROS scavenging. In addition, exogenous BK also inhibited p21 upregulation induced by H_2_O_2_ in H9C2 cells, so at least in part, we speculated that exogenous BK treatment inhibited ROS-mediated senescence process itself, but it still needs to be further elucidated.

In conclusion, we have demonstrated that exogenous BK administration markedly inhibited H9C2 cells DNA damage and subsequent senescence induced by H_2_O_2_ through reducing ROS generation and increasing ROS scavenging dependent on B2 receptor mediated NO release. Therefore, bradykinin B2 receptor may be a critical and promising therapeutic target for prevention and treatment in cardiomyocytes senescence.

## Supporting Information

Figure S1 **Effects of BK on p21 and B2 receptor expression in H9C2 cells treated with H_2_O_2_ and HOE-140.** H_2_O_2_ treatment significantly upregulated P21 expression and downregulated B2 receptor expression in H9C2 cells, and BK administration partly prevented the upregulation of P21 expression and downregulation of B2 receptor expression induced by H_2_O_2_. Importantly, HOE-140 totally abrogated the effects of BK on the regulation of p21 and B2 receptor expression. Bars represent means ±SEM (*P<0.05 vs control; ^#^ *P*<0.05 vs H_2_O_2_; ^&^ *P*<0.05 vs H_2_O_2_+BK; n = 3 experiments).
(JPG)

Figure S2 **Effects of BK on p21 and B2 receptor expression in H9C2 cells treated with H_2_O_2_ and L-NAME.** H_2_O_2_ treatment significantly upregulated P21 expression and downregulated B2 receptor expression in H9C2 cells, and BK administration partly prevented the upregulation of P21 expression and downregulation of B2 receptor expression induced by H_2_O_2_. Importantly, L-NAME totally abrogated the effects of BK on the regulation of p21 and B2 receptor expression. Bars represent means ±SEM (*P<0.05 vs control; ^#^ *P*<0.05 vs H_2_O_2_; ^&^ *P*<0.05 vs H_2_O_2_+BK; n = 3 experiments).
(JPG)
