# Supplementary figures and images for: Bradykinin Inhibits Oxidative Stress-Induced Cardiomyocytes Senescence via Regulating Redox State
Source: PLoS One. 2013 Oct 25;8(10):e77034. doi: 10.1371/journal.pone.0077034 (PMC3808370; doi:10.1371/journal.pone.0077034)

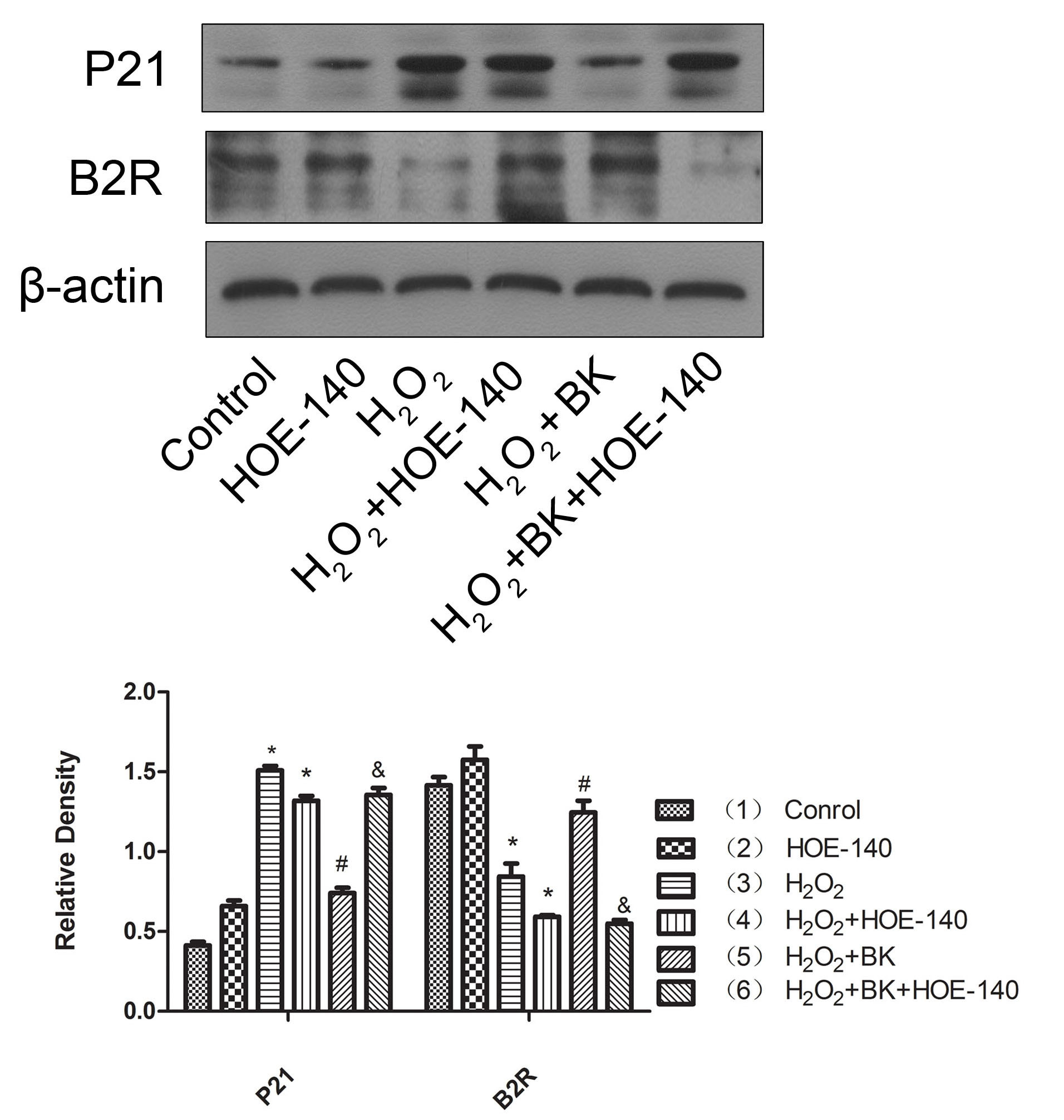

Supplement: Figure S1 — Effects of BK on p21 and B2 receptor expression in H9C2 cells treated with H2O2 and HOE-140. H2O2 treatment significantly upregulated P21 expression and downregulated B2 receptor expression in H9C2 cells, and BK administration partly prevented the upregulation of P21 expression and downregulation of B2 receptor expression induced by H2O2. Importantly, HOE-140 totally abrogated the effects of BK on the regulation of p21 and B2 receptor expression. Bars represent means ±SEM (*P<0.05 vs control; # P<0.05 vs H2O2; & P<0.05 vs H2O2+BK; n = 3 experiments). (JPG) [file pone.0077034.s001.jpg]

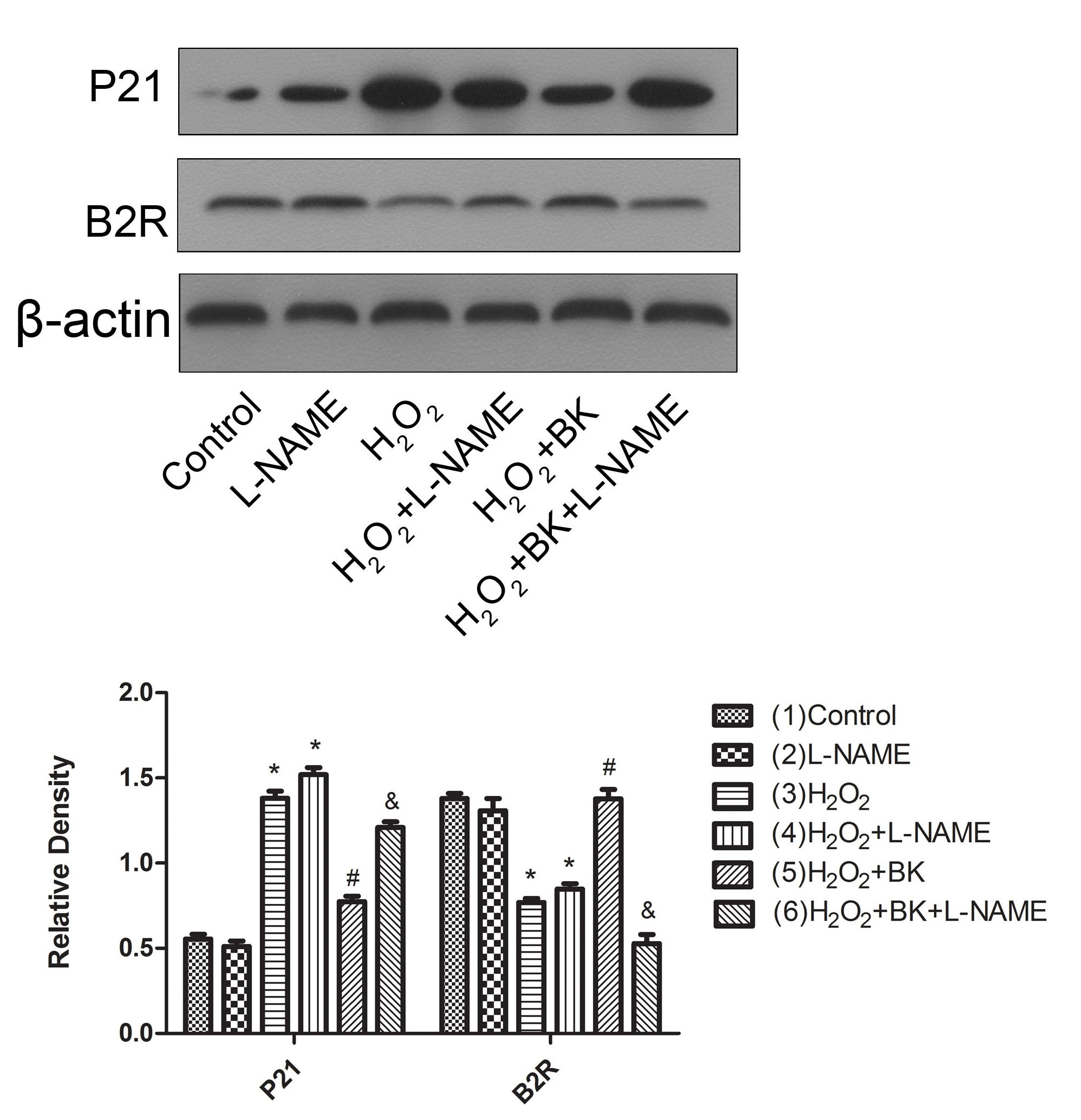

Supplement: Figure S2 — Effects of BK on p21 and B2 receptor expression in H9C2 cells treated with H2O2 and L-NAME. H2O2 treatment significantly upregulated P21 expression and downregulated B2 receptor expression in H9C2 cells, and BK administration partly prevented the upregulation of P21 expression and downregulation of B2 receptor expression induced by H2O2. Importantly, L-NAME totally abrogated the effects of BK on the regulation of p21 and B2 receptor expression. Bars represent means ±SEM (*P<0.05 vs control; # P<0.05 vs H2O2; & P<0.05 vs H2O2+BK; n = 3 experiments). (JPG) [file pone.0077034.s002.jpg]
